# Habitat Degradation and Seasonality Affect Physiological Stress Levels of *Eulemur collaris* in Littoral Forest Fragments

**DOI:** 10.1371/journal.pone.0107698

**Published:** 2014-09-17

**Authors:** Michela Balestri, Marta Barresi, Marco Campera, Valentina Serra, Jean Baptiste Ramanamanjato, Michael Heistermann, Giuseppe Donati

**Affiliations:** 1 Department of Social Sciences, Oxford Brookes University, Oxford, United Kingdom; 2 Department of Biology, University of Pisa, Pisa, Italy; 3 QIT Madagascar Minerals, Rio Tinto, Tolagnaro, Madagascar; 4 Endocrinology Laboratory, German Primate Center, Leibniz Institute for Primate Research, Goettingen, Germany; Midwestern University & Arizona State University, United States of America

## Abstract

The littoral forest on sandy soil is among the most threatened habitats in Madagascar and, as such, it represents a hot-spot within a conservation hot-spot. Assessing the health of the resident lemur fauna is not only critical for the long-term viability of these populations, but also necessary for the future re-habilitation of this unique habitat. Since the Endangered collared brown lemur, *Eulemur collaris*, is the largest seed disperser of the Malagasy south-eastern littoral forest its survival in this habitat is crucial. In this study we compared fecal glucocorticoid metabolite (fGCM) levels, a measure of physiological stress and potential early indicator of population health, between groups of collared brown lemurs living in a degraded forest fragment and groups occurring in a more preserved area. For this, we analysed 279 fecal samples collected year-round from 4 groups of collared brown lemurs using a validated 11-oxoetiocholanolone enzyme immunoassay and tested if fGCM levels were influenced by reproductive stages, phenological seasons, sex, and habitat degradation. The lemurs living in the degraded forest had significantly higher fGCM levels than those living in the more preserved area. In particular, the highest fGCM levels were found during the mating season in all animals and in females during gestation in the degraded forest. Since mating and gestation are both occurring during the lean season in the littoral forest, these results likely reflect a combination of ecological and reproductive pressures. Our findings provide a clear indication that habitat degradation has additive effects to the challenges found in the natural habitat. Since increased stress hormone output may have long-term negative effects on population health and reproduction, our data emphasize the need for and may add to the development of effective conservation plans for the species.

## Introduction

The term “stressor” is used to refer to any internal or external stimuli that perturb homeostasis of living organisms [1], [2]. There are several potential stressors for free-ranging animals. These can be natural, such as adverse climatic factors [3], high predation pressure [4], decrease in food availability [5], social aggression or competition [6], or anthropogenic, such as habitat degradation and fragmentation [7], [8], logging and hunting [9], and noise pollution [10].

In support of behavioural studies, hormonal studies have been recently used to assess animals' adaptability to such challenges and their welfare [11]–[13]. In vertebrates, stressors, depending on their severity, may cause a physiological response that entails an increase in glucocorticoid (i.e., a class of steroid hormones) secretion from the adrenal cortex [1], [14]. This response is considered adaptive in helping the animal to face critical periods that are threatening to homeostasis [13], [15], [16].

In spite of the positive short-term effects, the action of glucocorticoids may cause severe problems when animals are exposed to long-term (chronic) stressors [14], [17]. Among the most common problems caused by long-term elevated glucocorticoid levels are immune suppression, atrophy of tissue, reproductive suppression, gastric ulcers, and muscle wasting [18]–[21]. Furthermore, species with slow life histories, such as primates, may be particularly strongly impacted by lost reproductive opportunities resulting from chronic stress [22]. Since chronic stressors can lead to health risks [19], [23], high glucocorticoid levels are often assumed to indicate lower individual fitness or population viability [17]. However, this generalized view is debated, since recent studies suggest a complex relationship between glucocorticoids, stressors, and fitness [5], [24]–[26]. Despite the associated fitness costs, chronic stress has been proposed to evolve in species where it has an adaptive role in helping to face long-term stressors [13].

Reproductive stages may considerably affect glucocorticoid levels in vertebrates [27]. In the case of primates, physiological stress levels have been shown to increase in males during mating seasons as a consequence of high reproductive competition [28]–[34], but not in species where mate competition is low [22], [35], [36]. Females, on the other hand, have higher energetic demands during gestation and lactation and this may also lead to higher physiological stress [34], [37]–[41]. This seems to hold true despite the fact that, in primates, females have been shown to have lower energetic costs during reproductive stages than other mammals of similar size [42]–[44]. Furthermore, females are expected to have higher glucocorticoid levels during gestation than during lactation because of (1) placental release of corticotropin-releasing hormone which directly affects both fetal and maternal HPA-axis activity (reviewed by [45]), (2) increased synthesis of cortisol binding corticosteroid-binding globulin, (e.g. [46]) and (3) pregnancy-related increases in levels of estrogens (e.g. [47]).

Habitat disturbance has also been found to be associated with physiological stress in primates [48]–[51] and in other vertebrate species [52]–[55]. Anthropogenic disturbance is a widespread phenomenon in Madagascar where a large proportion of the original habitats have been lost [56], [57]. Forest disturbance due to anthropogenic pressure has been shown to reduce food availability and diversity, emphasizing the ecological unpredictability of the island [58]. In fact, Madagascar has been shown to differ from other primate habitats, due to its relatively unpredictable rainfall which leads to irregular fruiting patterns, making these environments challenging especially for frugivores [58], [59]. Despite this, recent studies have highlighted some degree of flexibility in frugivorous lemurs, which demonstrate a level of tolerance to habitat disturbance [60], [61]. Several frugivorous lemurs respond to habitat disturbance by integrating fallback food species and/or by shifting to a more folivorous diet [62], [63]. Additionally, activity patterns and ranging behaviour may be modified in order to maximize resource access or, alternatively, to conserve energy [64], [65].

The littoral forest of South-Eastern Madagascar is one of the most threatened habitats on the island with only a few hundred hectares of fragmented forest left [66], [67]. The Endangered collared brown lemur, *Eulemur collaris*, is the largest frugivorous lemur living in these forests, where it shows high social and ecological flexibility [61], [68]. However, a reduction of food availability and quality [61], [69] and an increase in parasite load [70] in the more disturbed areas indicate that these lemurs may be exposed to high physiological stress which, in turn, may lead to increased health risks. Since *Eulemur collaris* is also the largest seed disperser in the littoral forest [71] its survival in this habitat is crucial. Thus, assessing the impact of habitat disturbance on the stress physiology and welfare of this species is not only important for the long-term viability of the local populations, but also necessary for the future re-habilitation of this unique habitat [72].

In this paper, we examined how reproductive and phenological seasons, habitat degradation, and sex affect the physiological responses of collared brown lemurs. This response was investigated by comparing fecal glucocorticoid metabolite (fGCM) levels [73] between groups living in a degraded fragment (Mandena) and groups living in a more preserved fragment of littoral forest (Sainte Luce) in South-Eastern Madagascar. Fecal samples can be easily collected without disturbing the animal, thereby allowing frequent sampling, even over a long time period [74], thus they can be used as a powerful non-invasive measure of physiological stress levels in free-ranging animals [1], [12], [17], [37].

This study aims to elucidating whether the behavioural and ecological flexibility previously recorded in collared brown lemurs living in littoral forest fragments [61] may be sufficient to compensate for the non-optimal environment or whether the animals show increased signs of physiological stress.

Against this background we predicted higher fGCM levels:

1. In males during the mating season compared to other reproductive stages, because the mating season represents a period with pronounced reproductive competition in many species of primates;
2. In females during gestation and lactation compared to other reproductive stages, because of the expected higher energy demands;
3. During the lean season than during the season of abundance since the former is expected to be a time of food shortage in forest fragments due to low levels of fruit availability;
4. In the more degraded forest during stressful reproductive stages and the lean season, since anthropogenic disturbance may amplify fruit shortage and potentially increase exposure to climatic fluctuations and predators.

## Materials and Methods

### Ethics Statement

We conducted this study with the authorization of the Commission Tripartite of the Direction des Eaux et Forêts de Madagascar (Autorisation de recherche n.29/11/MEF/SG/DGF/DCB.SAP/SCB du 20/01/11) and this research was ethically approved by the University of Pisa (Animal Care and Use Board). We captured the adult individuals via cages, using banana slices as bait, and we rapidly anesthetized them with Zoletil 100 (5 mg/kg of tiletamine hydrochloride). All animals recovered from anesthesia within 1.5 hours and were subsequently released at the site of capture. The lemurs were followed until regaining full mobility in trees, and there were no injuries as a consequence of the captures.

### Study Sites and Subjects

The data were collected in two littoral forest areas from February 2011 to January 2012: Mandena (MAN) and Sainte Luce (STL) in South-East Madagascar. The Conservation Zone of MAN (24°57′S, 47°0′E) consists of two fragments of around 240 ha of degraded littoral forest [75]. The average canopy height in MAN is 8.9±SD 4.4 m [69]. The second study site, the littoral forest of STL (24°46′S, 47°10′E), around 30 km North of Fort Dauphin, is among the most intact littoral ecosystems in Madagascar and contains a very high diversity of vegetation [66]. The study area was located in a 252-ha-fragment of well-preserved littoral forest and swamp, 190 of which are included in the Conservation Zone [75]. The average canopy height in STL is 14.7±SD 4.3 m [69]. Previous botanical analyses illustrate that floristically MAN and STL represent the same habitat although structural differences indicate higher degradation in the former area [69]. Several lemur-focused studies confirm that MAN contains lower quality resources than STL in terms of fruit nutritional values and size of feeding trees [61], [65].

*Eulemur collaris* is a medium-sized lemur with body mass of 2.15±SD 0.25 kg and body length of 46.1±SD 2.6 cm [61]. These lemurs live in multi-male multi-female groups and show no clear dominance of one sex [61], [76]. In this study we analysed hormonal data collected from all adult individuals of four different groups (n = 22): two groups in MAN (group AB and group C), and two groups in STL (group A and group B).

To ensure continuous observations of the groups, four animals (one for each group) were captured and equipped with radio-collars in order to monitor them via the use of radio-telemetry (Biotrack). Collection of fecal samples began approximately one month after capturing the animals to minimize the risk that fGCM levels were influenced by the capture event itself. Individuals were identified via collars as well as individual characteristics such as age, sex, size, canine length, tail shape, fur colour, and other distinctive traits.

### Fecal sample collection and GC analysis

Each habituated group was followed four days (from 6 a.m. to 6 p.m.) per month in order to collect fecal samples and behavioral observations. A total of 279 fecal samples were collected from 22 subjects (mean per individual: 12.7±SE 0.3; range: 2–25). Each individual was sampled every 18.0±SE 1.9 days (range: 8.3–44.3 days). The samples were collected immediately after defecation. Site, group, date, time, and identity of the donor were recorded. Fecal samples were preserved in 10 ml tubes with 96% ethanol and stored at room temperature for 7–12 months before further processing for hormone analysis [77].

We collected 12 additional fecal samples to evaluate possible degradation of fGCM concentrations over one-year storage as reported for other species [78], [79]. For this, each fecal sample was divided into three aliquots and stored in ethanol as described above. Aliquots were kept at the field station at ambient temperature before being processed (see below), thus simulating the conditions under which the study samples were collected and stored. The first aliquot was extracted after 3 months, while the other two aliquots were extracted after 6 and 12 months, respectively, in order to match the longest storage time study samples were stored in ethanol. Fecal extracts were stored at −20°C before the final hormone analysis. The results showed no significant effect of storage duration on fGCM levels (RM ANOVA: Storage effect: F_2,22_ = 1.44, p = 0.258), a finding in line with what was found in *Propithecus verreauxi* [32] and in *Eulemur rufifrons* [33]. Thus, there was no indication that variation in storage duration biased our hormone data.

Fecal samples for hormone analyses were homogenized in their original ethanolic solvent by mechanical squashing of the fecal pellets with a metal stick. The ethanolic fecal suspension (including a 2-mL ethanol rinse) was decanted into a 50-mL propylene tube, and steroids were extracted by vortexing for 15 min. Following centrifugation at 3000 rpm for 10 min the supernatant was decanted, the exact volume recorded and stored at −20°C until analysis [77]. The remaining fecal pellets were dried in an oven to a constant weight and the dry weight of individual samples was determined in order to compensate for differences in fecal fibers and water content [80].

Fecal extracts were analyzed for immunoreactive 11oxoetiocholanolone (3α,11oxo-CM), a group specific measurement of 5-reduced cortisol metabolites with a 3α,11oxo-structure [81]. The assay has been successfully applied to monitor adrenocortical activity and glucocorticoid output from fecal samples in various primate species (e.g. [81]), including other species of lemurs [32], [82]. It has also been used successfully to monitor physiological stress in the redfronted lemur (*Eulemur rufifrons* [33]), a species closely related to the collared brown lemur. We used reverse-phase high pressure liquid chromatography analysis (HPLC) to characterize the immunoreactive metabolites measured by the 11oxoetiocholanolone EIA. HPLC was carried out as described by Heistermann et al. [81]. To evaluate possible sex differences in 11oxoetiocholanolone immunoreactivity profiles, we performed HPLC on both a male and a female sample. HPLC also allowed us to evaluate whether certain fecal androgens, which could potentially be detected by antibodies raised against cortisol metabolites [81], [83], were co-measured by the 11oxoetiocholanolone EIA.

**Figure 1:**
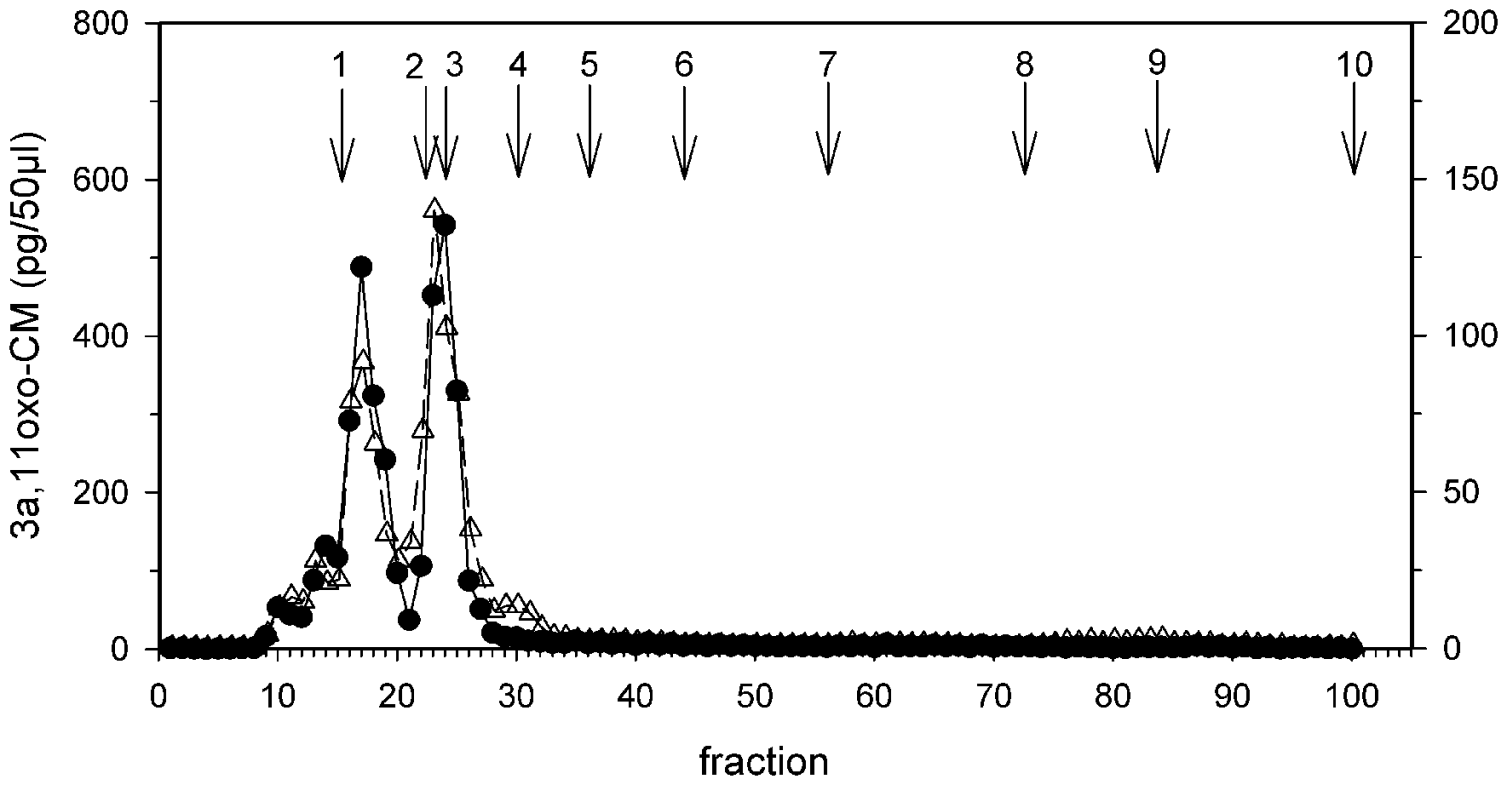
HPLC profiles of *Eulemur collaris*. HPLC profiles of immunoreactivity detected with the 3α,11oxo-CM assay in a male (black circles) and female (white triangles) fecal sample extract. Arrows indicate elution positions of reference standards (1) cortisol (fraction 15), (2) corticosterone (22), (3) 11β-hydroxyetiocholanolone (23/24), (4) 11-oxoetiocholanolone (29), (5) βandostrane-3,11,17-trione (36), (6) testosterone (44), (7) androstendione, dehydroepiandrosterone (55/56), (8) epiandrosterone, 5β-DHT, 5β-androstrane-3β-ol-17-one (72), (9) 5β-androstrane-3α-ol-17-one (82/83), (10) androsterone (100).

HPLC analysis indicated that almost all immunoreactivity was detected as distinct peaks between fractions 9 and 31 - positions where cortisol metabolites in our HPLC system elute (Fig. 1) [81]. The similarity between HPLC glucocorticoid immunoreactivity profiles from the collared brown lemur samples and those derived from fecal samples of other primate species [81], including the redfronted lemur [33], strongly suggests that the11oxoetiocholanolone assay is reliable in detecting glucocorticoid output in our study species. In this respect, the presence of only small amounts of immunoreactivity measured after fraction 40 (positions where certain potentially cross-reacting androgen metabolites elute [81]) suggests a low degree of co-measurement of these androgens in our assay (Fig. 1). Furthermore, HPLC profiles were almost identical between the male and female sample in terms of both number and elution position (i.e. characteristic) of metabolites measured, indicating that the 11oxoetiocholanolone assay measures the same immunoreactive compounds in both males and females.

For measurement of 3a,11 oxo-CM levels, fecal extracts were diluted (1∶20 to 1∶600 depending on concentration) with assay buffer (0.04 M PBS, pH 7.2) and duplicate 50 µl aliquots were measured by microtiterplate EIA along with 50 µl aliquots of reference standard in doubling dilutions over the range of 1.02–125 pg [81]. Briefly, following incubation of the plates overnight at 4°C, the plates were washed three times and incubated with 150 µl streptavidin–peroxidase (HRP) for 30 min in the dark at room temperature after which (following a second washing step) 150 µl of HRP-substrate solution was added to each well. Following substrate incubation (45 min), the enzyme reaction was stopped with 50 µl 2 M H_2_SO_4_ to each well and absorbance was measured at 450 nm (reference 630 nm) on a plate reader. Sensitivity of the assay was 3 pg. Serial dilutions of fecal extracts from samples of different animals gave displacement curves parallel to that obtained for the standard (*t*-test for difference in slopes between sample dilution curve and standard curve: *t*_11_ = 0.669, p = 0.517). Intra-assay coefficients of variation (CV) for low- and high value quality controls were 7.7% (n = 16) and 6.9% (n = 16), respectively. Respective figures for inter-assay CV values were 10.3% (n = 20) and 14.5% (n = 20). All hormone levels reported are expressed as ng/g dry fecal mass.

### Data analyses

In order to evaluate the effect of food availability on fGCM levels, we distinguished between a lean season (April–October) and a season of food abundance (November–March). The two seasons were distinguished on the basis of previous multi-annual studies in STL [84] and phenological data collected in MAN during our study period [65].

In order to evaluate the effect of the reproductive stages, we distinguished between four main stages: mating (May to mid-June), gestation (mid-June to September), lactation (October to December), and non-reproductive (January to April) [85].

All 279 fecal samples collected from the 22 adult individuals were used in the analyses. All adult females gave birth during the study period. Statistical comparisons were conducted using a General Linear Mixed Model (GLMM) with reproductive stages (nested in phenological seasons), sites, and sexes as fixed factors, and individuals as a random factor. Both main effects and two-way interaction effects were evaluated in the model. We controlled for the time of the day (morning or afternoon) when each sample was collected by including it in the model as fixed factor, since it has been shown to potentially affect fGCM levels [41], [86]. In fact, the fGCM levels were higher during the afternoon (median: 766.5 ng/g, quartiles: 438.5–1439.8 ng/g, n = 181) than during the morning (median: 600.3 ng/g, quartiles: 424.9–1013.3 ng/g, n = 98) (GLMM, Time of day: F_1,265_ = 6.14, P = 0.014). We also included in the model the sample weight as a covariate (fixed effect) to control for the potential effects of the fecal mass on hormone concentrations [86]. The sample weight was negatively correlated with the fGCM levels (GLMM, Weight: F_1,265_ = 67.15, p<0.001).

We used Duncan's tests as post hoc analyses. We tested for normal distribution of residuals (Kolmogorov-Smirnov test) and equality of variances (Levene's test) as underlying assumptions of the GLMM. Residual values of fGCM levels were not normally distributed and therefore the data were ln transformed. We performed all tests with SPSS 21.0 considering p<0.05 as threshold of significance.

## Results

**Figure 2:**
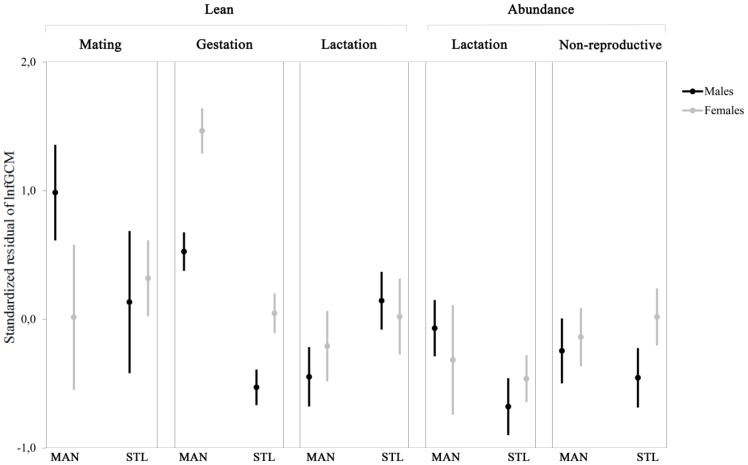
Fecal glucocorticoid metabolite levels of *Eulemur collaris* over the study period. The figure shows standardized residuals of lnfGCM after controlling for the sample weight. MAN: Mandena, STL: Sainte Luce. Lean: May–October 2011, Abundance: February–April 2011 and November 2011–January 2012. Mating: 1^st^ May–15^th^ July, Gestation: 16^th^ July–30^th^ September, Lactation: 1^st^ October–31^st^ December, Non-reproductive: 1^st^ January–30^th^ April. Values are means and standard errors.

The fGCM levels excreted by the 22 adult individuals (Fig. 2) did not differ between males (median: 666.1 ng/g, quartiles: 361.4–1318.5 ng/g, n = 164) and females (median: 740.1 ng/g, quartiles: 487.2–1309.1 ng/g, n = 115) (GLMM, Sex: F_1,265_ = 1.54, p = 0.216).

The fGCM levels differed between the four reproductive stages (GLMM, Reproductive stage: F_3,265_ = 2.62, p = 0.048), with a median of 1307.6 ng/g (quartiles: 710.9–2291.2 ng/g, n = 19) during mating, 766.5 ng/g (quartiles: 445.2–1473.1 ng/g, n = 111) during gestation stage, 612.4 ng/g (quartiles: 358.6–1132.4 ng/g, n = 115) during lactation stage, and 638.7 ng/g (quartiles: 474.6–990.3 ng/g, n = 34) during the non-reproductive stage. The fGCM levels during the mating season were higher than during gestation (Duncan: p = 0.003), lactation (p<0.001), and non-reproductive (p<0.001) stages.

The lemurs also had higher fGCM values during the lean season (median: 803.5 ng/g, quartiles: 463.3–1473.8 ng/g, n = 180) than during the season of food abundance (median: 569.3 ng/g, quartiles: 355.0–1002.0 ng/g, n = 99) (GLMM, Phenological season: F_1,265_ = 12.72, p<0.001).

The fGCM levels were higher in MAN (median: 870.2 ng/g, quartiles: 546.3–1714.9 ng/g, n = 126) than in STL (median: 590.9 ng/g, quartiles: 401.1–1014.6 ng/g, n = 153) (GLMM, Site: F_1,265_ = 20.27, p<0.001).A different pattern was found between the two sexes during the four reproductive stages (GLMM, Sex* Reproductive stage: F_3,265_ = 2.95, p = 0.033). In particular, males during the mating season had higher fGCM levels than during the periods corresponding to female gestation (Duncan: p = 0.004), lactation (p<0.001), and non-reproductive stage (p<0.001). Females during lactation had lower fGCM levels than during mating (p = 0.008) and gestation (p = 0.030). Interaction effects indicated that the two sexes did not show a different pattern between the two sites (GLMM, Sex*Site: F_1,265_ = 0.10, p = 0.755) and between the two phenological seasons (GLMM, Sex*Phenological season: F_1,265_ = 0.11, p = 0.742) (Table 1 and Table 2).

**Table 1:** Fecal glucocorticoid metabolite levels (ng/g) in males of *Eulemur collaris* over the study period.

	MAT (11)	GES (68)	LAC (68)	NRE (17)	LEA (109)	ABU (55)	Total
MAN	**1856**	**1227**	**673**	**572**	**1015**	**601**	**837**
(82)	1308–3151	683–1822	355–1006	376–842	597–1679	376–1011	500–1440
STL	**1019**	**430**	**844**	**571**	**587**	**486**	**498**
(82)	602–1618	298–590	300–1495	329–990	340–984	282–1002	303–1002
Total	**1564**	**611**	**704**	**572**	**770**	**512**	
	770–3151	412–1314	310–1280	376–842	428–1435	306–1011	

Values are medians and quartiles.

MAN: Mandena (more degraded site); STL: Sainte Luce (more preserved site); MAT: mating; GES: gestation; LAC: lactation; NRE: non reproductive; LEA: lean season; ABU: period of food abundance.

**Table 2:** Fecal glucocorticoid metabolite levels (ng/g) in females of *Eulemur collaris* over the study period.

	MAT (11)	GES (68)	LAC (68)	NRE (17)	LEA (109)	ABU (55)	Total
MAN	**711**	**1981**	**593**	**822**	**1621**	**561**	**1012**
(82)	676–2291	1621–2683	386–846	397–982	679–2462	386–982	600–1981
STL	**1265**	**713**	**588**	**813**	**729**	**630**	**713**
(82)	1091–1580	505–882	439–1060	566–1168	507–1060	475–931	483–1035
Total	**1178**	**976**	**588**	**822**	**896**	**609**	
	694–1936	596–1844	421–1013	559–1000	551–1621	392–957	

Values are medians and quartiles.

MAN: Mandena (more degraded site); STL: Sainte Luce (more preserved site); MAT: mating; GES: gestation; LAC: lactation; NRE: non reproductive; LEA: lean season; ABU: period of food abundance.

Results of pair-wise comparisons of mean differences between sites, reproductive stages, and phenological seasons for ln transformed fGCM values in males and females are shown in Table 3 and Table 4, respectively.

**Table 3:** *P* values of pair-wise comparisons of mean differences between sites in ln transformed fGCM values across different reproductive stages in males of *Eulemur collaris* (Duncan post-hoc).

Site		MAN	MAN	MAN	MAN	MAN	STL	STL	STL	STL	STL
	Stage	MAT-L	GES-L	LAC-L	LAC-A	NRE-A	MAT-L	GES-L	LAC-L	LAC-A	NRE-A
MAN	MAT-L	-	-	-	-	-	-	-	-	-	-
MAN	GES-L	0,12	-	-	-	-	-	-	-	-	-
MAN	LAC-L	**0,00**	0,06	-	-	-	-	-	-	-	-
MAN	LAC-A	**0,01**	0,23	0,46	-	-	-	-	-	-	-
MAN	NRE-A	**0,00**	0,11	0,74	0,65	-	-	-	-	-	-
STL	MAT-L	0,07	0,71	0,12	0,37	0,21	-	-	-	-	-
STL	GES-L	**0,00**	0,02	0,58	0,23	0,41	**0,04**	-	-	-	-
STL	LAC-L	0,10	0,85	0,08	0,29	0,15	0,82	**0,03**	-	-	-
STL	LAC-A	**0,00**	**0,04**	0,84	0,37	0,62	0,09	0,70	0,06	-	-
STL	NRE-A	**0,00**	0,07	0,92	0,50	0,79	0,14	0,55	0,10	0,78	-

Median sample size: 15 (range: 5–36).

MAN: Mandena (more degraded site); STL: Sainte Luce (more preserved site); MAT: mating; GES: gestation; LAC: lactation; NRE: non reproductive; L: lean period; A: period of food abundance.

**Table 4:** P values of pair-wise comparisons of mean differences between sites in ln transformed fGCM values across different reproductive stages in females of *Eulemur collaris* (Duncan post-hoc).

Site		MAN	MAN	MAN	MAN	MAN	STL	STL	STL	STL	STL
	Stage	MAT-L	GES-L	LAC-L	LAC-A	NRE-A	MAT-L	GES-L	LAC-L	LAC-A	NRE-A
MAN	MAT-L	-	-	-	-	-	-	-	-	-	-
MAN	GES-L	**0,07**	-	-	-	-	-	-	-	-	-
MAN	LAC-L	0,15	**0,00**	-	-	-	-	-	-	-	-
MAN	LAC-A	**0,08**	**0,00**	0,73	-	-	-	-	-	-	-
MAN	NRE-A	0,23	**0,00**	0,77	0,55	-	-	-	-	-	-
STL	MAT-L	0,60	0,17	0,06	**0,03**	0,10	-	-	-	-	-
STL	GES-L	0,23	**0,00**	0,78	0,56	0,97	0,10	-	-	-	-
STL	LAC-L	0,64	**0,03**	0,31	0,19	0,42	0,35	0,42	-	-	-
STL	LAC-A	0,11	**0,00**	0,84	0,86	0,65	**0,04**	0,66	0,24	-	-
STL	NRE-A	0,65	**0,03**	0,30	0,19	0,42	0,36	0,42	0,98	0,24	-

Median sample size: 9 (range: 3–25).

MAN: Mandena (more degraded site); STL: Sainte Luce (more preserved site); MAT: mating; GES: gestation; LAC: lactation; NRE: non reproductive; L: lean period; A: period of food abundance.

## Discussion

Consistent with our predictions, the highest fGCM values were found in females during the gestation period in the degraded forest of MAN and in males during the mating season in both sites. Phenological season also played a role in shaping the fGCM output with higher values exhibited during the lean season, whilst sex had no significant effect. Fecal glucocorticoid metabolite levels were higher during the afternoon rather than during the morning, a characteristic of nocturnal animals which, from a chronobiological perspective, *Eulemur* species belong to [87]. The covariate sample weight also significantly influenced the fGCM output.

As predicted, in males we found higher fGCM levels during the mating season than during the other reproductive stages. Mating season appears to be a stressful period also for male mouse lemurs (*Microcebus murinus* [28]), male ring-tailed lemurs (*Lemur catta* [31], [34]), male sifakas (*Propithecus verreauxi* [32]), and male red-fronted lemurs (*Eulemur rufifrons* [33]). In fact, during this study, collared brown lemur males showed higher aggression rates during the mating season as compared to the other reproductive stages (Serra et al., unpublished data). It is reasonable to assume that the higher fGCM levels found during this time of the year may be related to a general increase of aggression rates and high reproductive competition [88]. Increased aggression rates have been shown to affect fGCM levels in males of other primate species, such as Eastern chimpanzees (*Pan troglodytes* [29]), chacma baboons (*Papio ursinus* [30]), Verreaux's sifakas (*Propithecus verreauxi* [32]) and in males of other vertebrates (wolves, *Canis lupus*, [89]; bison bulls, *Bison bison*, [90]; American alligators, *Alligator mississippiensis*, [91]). Conversely, low physiological stress levels have been observed in species where mate competition is low, such as males of muriquis (*Brachyteles arachnoides*, [35]), tufted capuchin monkeys (*Cebus apella*, [36]), and red-bellied lemurs (*Eulemur rubriventer* [22]), while no differences were found between mating and non-mating seasons in male rhesus macaques (*Macaca mulatta*, [92]).

In our study, we also found high fGCM levels in females during the mating season, suggesting that this period may also be stressful for them. This is in accordance with findings on other primates (e.g. South-american squirrel monkeys, *Saimiri sciureus*, [93], [94]) and mammals (e.g. giant pandas, *Ailuropoda melanoleuca*, [95], [96]) where the GC elevation has been attributed to the effects of ovarian cycling and general anxiousness. However, we must consider that our results indicate a very high variability within the mating season, which may be due to two confounding factors. Firstly, we had a small sample size due to the very short mating season [58], and we may have accidentally included samples belonging to the previous and/or the subsequent reproductive stage. Secondly, individuals may have different levels of physiological stress depending on their dominance status [97].

By far, the highest fGCM levels were found in females in the more degraded site during gestation (see Fig. 2). It is well known that gestation represents a serious challenge for females, as during this period they have increased energetic demands, as previously shown for females of ring-tailed lemurs (*Lemur catta*, [37]), white faced capuchins (*Cebus capucinus*, [40]), and other mammalian species (little brown myotis, *Myotis lucifugus*, [98]; red squirrels, *Tamiasciurus hudsonicus*, [99]). Higher glucocorticoid levels during gestation may not necessarily correspond to higher physiological stress, however, since placental hormones and fetal estrogens also stimulate cortisol production [100]. In the Malagasy littoral forest, the physiological increase in glucocorticoid levels due to gestation may be enhanced by the additional physiological stress due to the concomitant lean season [84]. However, the significantly lower fGCM levels found in the females inhabiting the less disturbed fragment during gestation strongly suggests that the degraded habitat conditions are largely responsible for this effect. Interestingly, during gestation in the degraded forest males had similar fGCM levels as the females within the same site. This supports the idea that habitat degradation and seasonal food availability override the potential effect of female reproductive state on fGCM levels. The fact that pregnant females showed relatively low levels of fGCM in the less disturbed area is also in line with previous studies showing that lemurs minimize maternal energetic investment during gestation [100].

Contrary to other studies, collared brown lemur females did not show high fGCM levels during lactation, a potentially stressful period for females due to the burden of infant carrying and maternal care (ring-tailed lemurs, *Lemur catta*, [34], [38], but see [101]; Assamese macaques, *Macaca assamensis*, [102]; rhesus macaques, *Macaca mulatta*, [39]). The lack of elevated fGCM levels in lactating females of collared brown lemurs may have been caused by the overriding effect of the concomitant increase in food availability. Similar results have been shown in *Lemur catta* at Beza Mahafaly which also showed low physiological stress levels during lactation [37]. In fact, lactation in collared brown lemurs is synchronized with the transition from the lean season to the season of food abundance, when young leaves and ripe fruits increase in their availability [56], [84], [103]. Additionally, primates produce some of the most dilute milk of all mammals [42] and, in particular, milk-producing costs for *Eulemur* species are among the lowest amongst primates [104].

As expected, we found that seasonality had a strong effect on fGCM output in collared brown lemurs. This was in the expected direction as we found higher fGCM values during the lean season, when fruit availability was low [65]. In primates physiological stress levels have been repeatedly found to be shaped by fruit availability (olive baboons, *Papio anubis*, [105]; ring-tailed lemurs, *Lemur catta*, [37], [38]; chimpanzees, *Pan troglodytes*, [29]; black howler monkeys, *Alouatta pigra*, [106]; yellow baboons, *Papio cynocephalus*, [107]; Mexican howler monkeys, *Alouatta palliata*, [51]) and periods of presumed nutritional stress (Eastern red colobus, *Procolobus rufomitratus*, [108]). In particular, in MAN, the percentage of tree species with ripe fruits averaged 4.4% during the mating season and the gestation period while it stands at 14.2% during lactation and non-reproductive stages [65]. Thus, low fruit availability seems to have an additive effect on reproductive stages and habitat degradation, and the combination of these three factors is likely to reflect fGCM output in our study species.

This study clearly indicates that levels of fGCM were higher for collared brown lemurs in the degraded forest fragment of MAN when compared to lemurs in the more preserved forest of STL, suggesting a higher level of physiological stress in animals living in disturbed areas. The most likely explanation for this difference may be found in the lower levels of food availability [65] and quality [61] recorded in MAN as compared to STL which may result in increased nutritional stress. Other stressors, such as a higher predation risk in the disturbed, more open MAN forest, may have contributed to the recorded difference. In support of this, the lemurs' primary predator, the fossa (*Cryptoprocta ferox*), was reported several times in MAN but not in STL over the last decade [109].

Previous studies show that collared brown lemurs in the degraded forest shape their ranging, feeding, and activity pattern to cope with a decrease in food abundance [61], [65]. In particular, lemurs in MAN during our study period had larger home ranges and traveled shorter daily distances than lemurs in STL [65]. Our results indicate that, although the collared brown lemurs seem to cope with habitat degradation by changing their behavioral ecology, living in a degraded forest area nevertheless increases physiological stress. This may have an effect on the long-term viability of the population. These effects may include higher vulnerability to diseases, reduced reproduction, and even a higher mortality rate [17]. Our finding of a higher parasite burden for the lemurs in MAN as compared to those in STL [70] is in accordance with this, and may indicate that elevated glucocorticoid levels do pose a health risk to the animals, although the cause-and-effect relationship between these two parameters is not entirely clear.

Our findings are in line with other recent studies which found that habitat degradation and fragmentation are associated with an increase in GC outputs in a variety of primates (Eastern red colobus, *Procolobus rufomitratus*, [108]; black howler monkeys, *Alouatta pigra*, [49]; Yucatan spider monkeys, *Ateles geoffroyi*, [50]; Mexican howler monkyes, *Alouatta palliata*, [51]; but see [9] for effect of logging and hunting on brown spider monkeys, *Ateles hybridus* and red howler monkeys, *Alouatta seniculus*) and other animal species (American redstarts, *Serophaga ruticilla*, [52]; spotted salamanders, *Ambystoma maculatum*, [53]; African savanna elephants, *Loxodonta africana*, [54]; agile antechinus, *Antechinus agilis*, [55]; see also [110] for effects of human disturbance on elks, *Cervus elaphus*, and wolves, *Canis lupus*). Conversely, the only previous study which compared lemurs' fGCs levels in disturbed and undisturbed habitats [22] showed opposite results. In the latter study, red-bellied lemurs (*Eulemur rubriventer*) in the undisturbed forest of Ranomafana showed higher fecal cortisol levels than those in the disturbed habitat during the lean period. This result might be explained by an attenuated response to prolonged stress to reduce costs of continued stress hormone production [14]. In fact, *Eulemur rubriventer* in the disturbed habitat showed higher infant mortality [22]. Conversely, lemurs in MAN have birth and mortality rates similar to those in STL [84] and in other more preserved forests [111]. Thus, lemurs in MAN do not give indications of an attenuated response to habitat degradation, but they do not seem to exhibit any clear negative effects at the population level. Furthermore, *Eulemur rubriventer* in Ranomafana relied on the exotic *Psidium cattleianum* in the disturbed area, and this might have shielded them during lean periods [22]. In fact, exotic fruits are known to sometimes provide a nutritionally higher resource and a longer temporal availability than native species [112]. Contrary to Tecot's study [22], collared brown lemurs do not seem to rely on exotic species in MAN [61] and this may also explain the high fGCM levels detected during the lean season.

In addition to Tecot [22], studies on other animals (bighorn sheep, *Ovis canadiensis* [113]; Canadian grizzly bears, *Ursus arctos*, [114]; African forest elephants, *Loxodonta cyclotis*, [115]; spotted salamanders, *Ambystoma maculatum*, [53]) found higher fGCM in undisturbed habitats. Thus, the hypothalamic–pituitary–adrenal axis response may differ between species [13] and the relationship between GC levels and stressors may be not so clear-cut.

Another possible stressor that may have influenced the higher fGCM levels found in lemurs living in MAN is their proximity to a mining site. The area of MAN in which the study took place is in fact very close to the machinery set up in the region to extract titanium deposits [116]. This may have exposed the lemurs to chronic stressors such as anthropogenic noise and light pollution. In particular, noise pollution may lead to behavioural changes [10] and to an increase in fGCM levels [117]. *Eulemur collaris* is a species known to shape its 24-h activity depending on luminosity [68], [118]. Hence, artificial light pollution may potentially alter the species behaviour. For example, human activities have been shown to alter the activity budget of bighorn sheep [113]. Further studies focusing on these aspects may give a clearer insight on the impact of mining on the area.

## Conclusions

By comparing fGCM levels in collared brown lemurs living in a degraded and in a more preserved forest fragment, we found higher fGCM levels occur in those individuals living in the former situation, which appears to be a stressful environment. Higher fGCM levels in disturbed habitats suggest that coping with a harsher environment has a cost of increased physiological stress in these lemurs. This study underlines the importance of physiological investigations to assess population health of threatened species and the potentially detrimental effect of habitat loss on animal welfare. Because of the paucity of studies comparing lemurs living in disturbed environments over the long-term, more research is urgently required to evaluate the consequences of chronic physiological stress on the highly threatened lemur populations.
